# Bioproduction of *N*^6^-benzyladenine *N*^9^-β-D-glucopyranoside using suspension cells of bamboo (*Phyllostachys nigra*)

**DOI:** 10.5511/plantbiotechnology.25.0507a

**Published:** 2025-12-25

**Authors:** Taiji Nomura, Karin Okazaki, Mikihisa Umehara, Yasuo Kato

**Affiliations:** 1 Biotechnology Research Center and Department of Biotechnology, Toyama Prefectural University, 5180 Kurokawa, Imizu, Toyama 939-0398, Japan; 2 Graduate School of Life Sciences, Toyo University, 48-1 Oka, Asaka, Saitama 351-8510, Japan

**Keywords:** bamboo, bioproduction, cultured plant cell, cytokinin, *N*^6^-benzyladenine glucoside

## Abstract

Suspension-cultured cells of a temperate bamboo species (*Phyllostachys nigra*) accumulate substantial amounts of hydroxycinnamic acid derivatives and lignin under culture conditions that promote xylogenesis. In our previous study, we found a metabolite specifically produced in bamboo cells cultured under lignification-inducing conditions in a medium containing *N*^6^-benzyladenine (BA), but the chemical structure was not elucidated. In this study, we purified and identified this compound as BA *N*^9^-β-D-glucopyranoside (BA-9G). Despite the presence of three nitrogen positions (*N*-3, *N*-7, and *N*-9) that may be glucosylated in the adenine moiety of BA, bamboo cells specifically produced BA-9G (i.e., without other glucoside types) when cells were cultured in the presence of BA. This finding suggests that bamboo cells possess a regio-specific *N*-glucosyltransferase for catalyzing cytokinin glucoside formation. The biological activity of BA-9G as a cytokinin was compared with that of BA on the basis of adventitious shoot formation on internodal segments of ipecac (*Carapichea ipecacuanha*) plants grown under in vitro conditions. The activity of BA-9G was more moderate than that of BA, but BA-9G was less cytotoxic than BA at a high concentration, suggesting that BA-9G may be useful as a plant growth regulator. The development of a viable system for the regio-specific bioproduction of BA-9G in bamboo cells may increase the availability of this highly expensive and rare cytokinin derivative.

Suspension-cultured cells of a temperate bamboo species (*Phyllostachys nigra*; Pn), which we established previously, are suitable for the bioproduction of phenylpropanoid-derived compounds via a rational metabolic-flow switching strategy (Kitaoka et al. 2020, 2021; Nomura et al. 2018; Ube et al. 2024). This strategy exploits Pn cell growth and metabolic features. Notably, the cell phase for rapid proliferation (inactive secondary metabolism) or active secondary metabolism (reduced proliferation) can easily be controlled by changing the culture medium (Nomura et al. 2013; Ogita et al. 2012). Pn cells cultured in half-strength (1/2) Murashige and Skoog (MS) medium (Murashige and Skoog 1962) supplemented with 10 µM *N*^6^-benzyladenine (BA), a synthetic cytokinin, accumulate substantial amounts of phenylpropanoid-derived metabolites, including hydroxycinnamoylputrescines and lignin (Nomura et al. 2013; Ogita et al. 2012).

During an earlier analysis of Pn cell extracts for secondary metabolite(s), we detected a compound that was specific to the extracts of Pn cells cultured in the presence of BA (designated as “Rt 7.0” on the basis of the high-performance liquid chromatography (HPLC) retention time; Nomura et al. 2013). We predicted that this compound (hereafter designated as compound 1) is a metabolite of BA, but the chemical structure was not determined. In this study, we first optimized culture conditions to enhance the production of compound 1 for its expedient isolation from cells, after which compound 1 was isolated and structurally characterized as a glucoside of BA with a limited availability. On the basis of the study results (e.g., comparative biological activity assay with BA), the metabolic and practical importance of this compound, which can be readily produced using Pn cells, is discussed.

BA was purchased from Fujifilm Wako Pure Chemical Corporation (Osaka, Japan). Authentic analytical standards of BA-glucosides, BA *N*^3^-β-D-glucopyranoside (BA-3G), BA *N*^7^-β-D-glucopyranoside (BA-7G), and BA *N*^9^-β-D-glucopyranoside (BA-9G), were purchased from Fujifilm Wako Pure Chemical Corporation, Olchemim (Olomouc, Czech Republic), and Toronto Research Chemicals (Toronto, ON, Canada), respectively.

Pn suspension cells, which are available from the RIKEN BioResource Research Center (no. rpc00047; https://web.brc.riken.jp/ (Accessed Mar 22, 2025)), were maintained and subcultured every 2 weeks as previously described (Nomura et al. 2013). Cell densities were determined according to the sedimented cell volume (SCV) as previously described (Ogita et al. 2011). To optimize culture conditions conducive to the production of compound 1, subcultured Pn suspension cells were transferred to the following fresh medium with an initial cell density of 5, 10, or 20% SCV: 1/2 MS medium containing 3% (w/v) sucrose and 1, 10, 100, or 250 µM BA. Cells were cultured, with samples collected every 2 days for 14 days as previously described (Nomura et al. 2013).

To analyze Pn cell extracts for compound 1, cells (approximately 100 mg fresh weight; FW) were mixed with 10 volumes of MeOH containing 2% (v/v) AcOH for a 10-min ultrasonication at room temperature. The resulting extract was centrifuged (21,500×g, 10 min, 4°C), and the supernatant was analyzed using a reversed-phase HPLC system as previously described (Nomura et al. 2013). For the HPLC analysis of the culture medium, the filtrate obtained after suspension-cultured cells were collected on filter paper was passed through a Millex-HV filter (0.45 µm; Merck, Darmstadt, Germany). The filtrate was analyzed by HPLC.

We first optimized Pn cell culture conditions for the high-level production of compound 1. Pn suspension cells were cultured in the presence of different concentrations of BA with an initial cell density of 5% SCV. Cell growth levels were similar between 1 µM and 10 µM BA (original concentration; Nomura et al. 2013), but decreased substantially at BA concentrations of 100 µM and 250 µM (Supplementary Figure S1A). Although we also cultured Pn suspension cells in the presence of 500 µM BA, the cells became bleached and were non-viable, indicating that the BA concentration was excessive for the cells. The production of compound 1 increased as the concentration of BA added to the culture medium increased (Supplementary Figure S1B), indicating that Pn cells can produce compound 1 at BA concentrations up to 250 µM, despite the suppressed cell growth at high BA concentrations.

Considering these results, we next examined the effects of the initial cell density on the production of compound 1 in culture medium in which the initial BA concentration was 250 µM. As shown in Supplementary Figure S2A, cell growth recovered when the initial cell density was 20% SCV, suggesting that Pn cells metabolized BA to a less cytotoxic form (i.e., compound 1). In fact, increases in the initial cell density accelerated the decrease in BA levels in the culture medium (Supplementary Figure S2B). The compound 1 content per cell FW started to increase immediately after starting the culture (Supplementary Figure S2C). By multiplying the compound 1 content by the cell FW at each collection time point, the highest production titer per liter culture was obtained on day 4 for the culture with an initial cell density of 20% SCV (Supplementary Figure S2D), which coincided with the complete consumption of BA in the culture medium (Supplementary Figure S2B). On the basis of these results, we decided to culture Pn cells for 4 days with an initial cell density of 20% SCV in the presence of 250 µM BA for the expedient isolation of compound 1 (Supplementary Figure S3).

We purified 122 mg compound 1 from Pn suspension cells (187 g FW) obtained from a 2.3-l culture under optimized conditions according to the procedures described in Supplementary Methods. The NMR spectra of compound 1 were recorded using an AVANCE 400 spectrometer (Bruker, Karlsruhe, Germany), with a mixture comprising 0.5 ml (CD_3_)_2_SO and 0.1 ml D_2_O (for ^1^H-NMR) or (CD_3_)_2_SO (for ^13^C-NMR) serving as the solvent. The UV spectrum was measured using a U-2000 spectrophotometer (Hitachi, Tokyo, Japan), with H_2_O serving as the solvent. The high-resolution electrospray ionization time-of-flight mass spectrometry (HR-ESI-TOF-MS) spectrum was obtained using a micrOTOF focus spectrometer (Bruker) and a published direct infusion method (Nomura and Kato 2020). The spectral properties of purified compound 1 were as follows: HR-ESI-TOF-MS (Supplementary Figure S4) (positive) *m*/*z* (relative intensity) 388.1634 [M+H]^+^ (100) (calcd. for C_18_H_22_N_5_O_5_^+^, 388.1615), *m*/*z* 410.1453 [M+Na]^+^ (47) (calcd. for C_18_H_21_N_5_O_5_Na^+^, 410.1435); UV (H_2_O) λ_max_ (logε) 268 nm (4.28); ^1^H-NMR (400 MHz, (CD_3_)_2_SO 0.5 ml + D_2_O 0.1 ml, Supplementary Figure S5A): δ (ppm) 3.32–4.03 (6H, m, H-2″, 3″, 4″, 5″, 6″), 4.74 (2H, brs, H-7′), 5.44 (1H, d, *J*=9.4 Hz, H-1″), 7.21–7.37 (5H, m, H-2′, 3′, 4′, 5′, 6′), 8.22 (1H, s, H-8), 8.33 (1H, s, H-2); ^13^C-NMR (100 MHz, (CD_3_)_2_SO, Supplementary Figure S5B): δ (ppm) 43.3 (C-7′), 61.4 (C-6″), 70.3 (C-4″), 71.7 (C-2″), 77.7 (C-5″), 80.5 (C-3″), 83.3 (C-1″), 119.5 (C-5), 127.0 (C-4′), 127.5 (C-2′, 6′), 128.7 (C-3′, 5′), 140.2 (C-1′), 140.6 (C-8), 149.7 (C-4), 153.0 (C-2), 154.9 (C-6).

The molecular formula (C_18_H_21_N_5_O_5_) and molecular weight (387) determined on the basis of the HR-ESI-TOF-MS data (Supplementary Figure S4) indicated that compound 1 is a glucoside of BA. Glucosylation of cytokinins, *N*^6^-substituted adenine derivatives, occurs at the hydroxy group of the *N*^6^-substituent (i.e., hydroxylated isoprenoid type) and at the *N*^3^, *N*^7^, or *N*^9^ position of the purine moiety (Chen et al. 2021). Because BA does not have a glucosylation site on the *N*^6^-benzyl moiety, compound 1 was predicted to be one of the three *N*-glucosides. Thus, compound 1 was analyzed by HPLC for a comparison of its retention time with that of the authentic *N*-glucosides of BA (BA-3G, BA-7G, and BA-9G), which indicated that compound 1 is BA-9G (Supplementary Figure S6). Moreover, in the ^1^H-NMR spectrum of compound 1, the doublet signal of an anomeric proton (H-1″) at 5.44 ppm (*J*=9.4 Hz) reflected the β-configuration of C-1″. Considered together, these findings suggest that compound 1 is *N*^6^-benzyladenine *N*^9^-β-D-glucopyranoside (BA-9G, Figure 1). The spectral data of compound 1 were in accordance with published data (Cowley et al. 1978; Duke and MacLeod 1978; Hashizume and Yoshida 1976).

**Figure 1:**
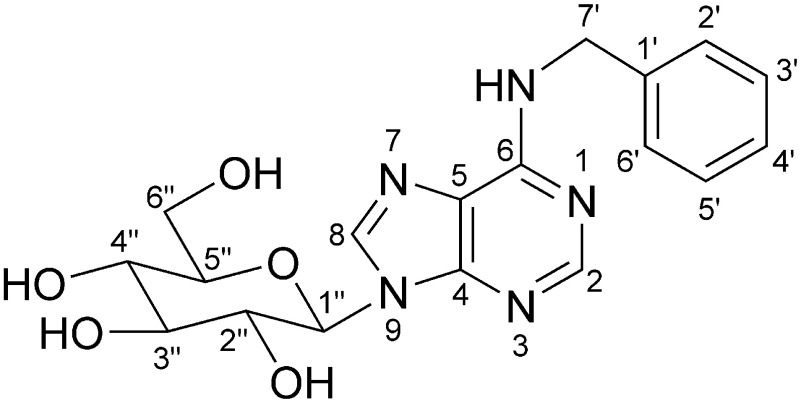
Figure 1. Chemical structure of *N*^6^-benzyladenine *N*^9^-β-D-glucopyranoside (BA-9G).

We previously demonstrated that adding 10 µM BA to the culture medium promotes the xylogenesis of Pn cells via the accumulation of lignin (Nomura et al. 2013; Ogita et al. 2012). To examine whether the same phenomenon is observed when BA-9G is added, Pn cells were cultured in the presence of 10 µM BA-9G with an initial cell density of 5% SCV. As shown in Figure 2A, Pn cells cultured in medium containing 10 µM BA-9G started to proliferate earlier than those cultured in medium supplemented with 10 µM BA. This is likely because BA-9G is less cytotoxic or biologically active than BA. When BA was added to the medium, the BA-9G content in cells peaked on day 2 (Figure 2B), which was consistent with the complete consumption of BA in the culture medium in 2 days (Figure 2C). The addition of 10 µM BA-9G to the culture medium resulted in a gradual increase in the BA-9G content in cells (Figure 2B). Moreover, the BA-9G content in the culture medium gradually decreased, but was not completely consumed during the 14-day culture period (Figure 2C). BA-9G was incorporated into cells less efficiently than BA likely because BA-9G is more hydrophilic than BA. Staining cells with a phloroglucinol–HCl reagent indicated that BA-9G can also promote Pn cell lignification (Supplementary Figure S7). Although lignification appeared to be slightly delayed under the BA-9G condition, the extent of lignification (i.e., intensity of the red staining) was similar between the BA and BA-9G conditions.

**Figure 2:**
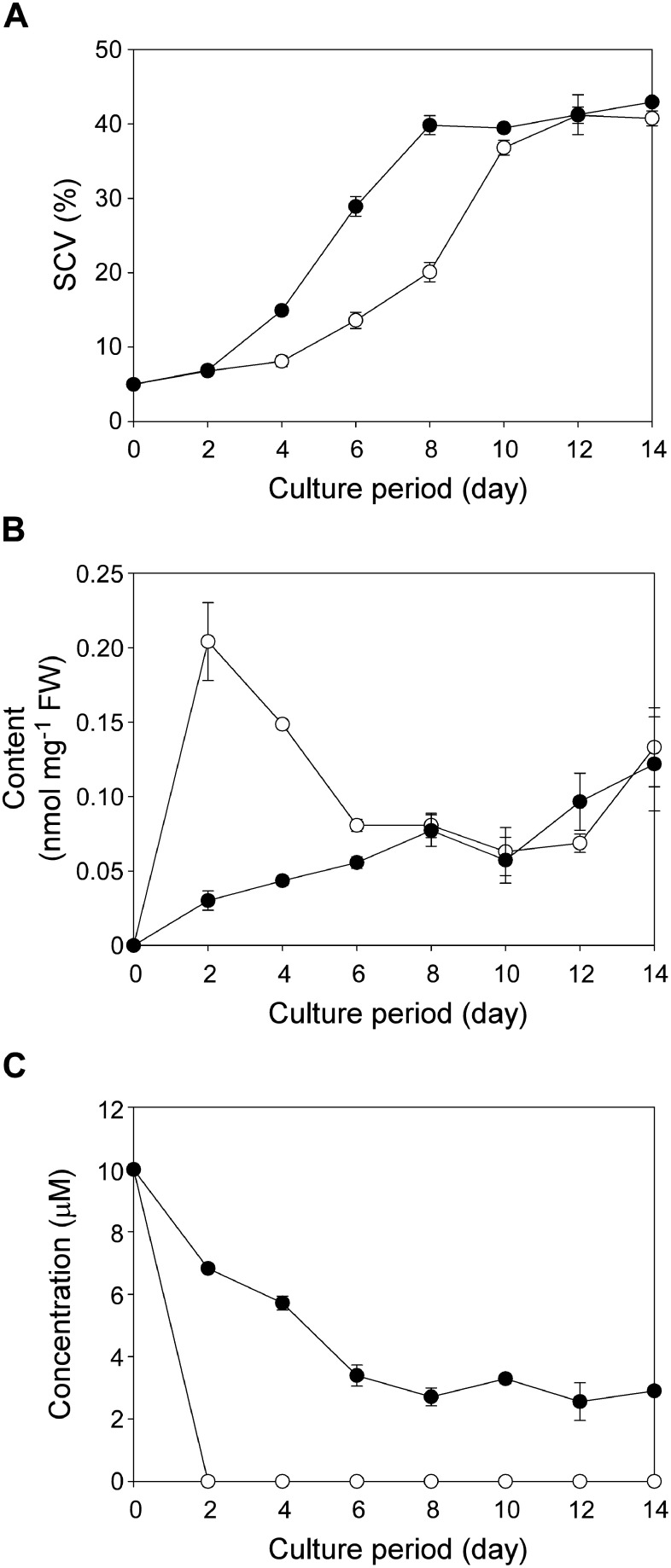
Figure 2. Time-course changes in cell proliferation and the BA-9G content in Pn suspension cells cultured in medium supplemented with BA or BA-9G. (A) Changes in SCVs with an initial cell density of 5% SCV (mean±SD, *n*=3). (B) Changes in the BA-9G content in cell extracts (mean±SD, *n*=3). (C) Changes in the BA/BA-9G concentration in the culture medium (mean±SD, *n*=3). Cells were cultured in medium supplemented with 10 µM BA (empty circles) or 10 µM BA-9G (filled circles).

We next examined the biological activity of BA-9G as a cytokinin using the adventitious shoot formation system involving cultured internodal segments of ipecac (*Carapichea ipecacuanha*) (Okazaki et al. 2025). These segments form adventitious shoots under phytohormone-free culture conditions, but shoot formation is biased toward the apical region because of differences in the endogenous cytokinin/auxin proportions between the apical and basal regions, with cytokinin/auxin proportions higher and lower in the apical and basal regions, respectively (Koike et al. 2017). Thus, this internode culture system is considered to be appropriate for evaluating the biological activity of exogenously applied cytokinins. The assay was performed as previously described (Okazaki et al. 2025). Briefly, 2.5-mm internodal segments were cut from sterile ipecac plants cultured in vitro. The segments were placed on B5 medium containing 0.1–10 µM BA-9G or BA and solidified with 0.8% (w/v) agar in a Petri dish (90 mm × 20 mm), which was then incubated at 24°C under a 14-h light/10-h dark photoperiod (10–20 µmol photons m^−2^ s^−1^). After culturing the internodal segments for 5 weeks, the number of adventitious shoots (longer than 0.3 mm) was determined for the apical and basal halves of each segment. As shown in Figure 3, adventitious shoots formed mainly in the apical region of internodal segments. When these segments were cultured in the presence of BA, the number of adventitious shoots increased as the BA concentration increased up to 1 µM, but adventitious shoots did not form at 10 µM. By contrast, a treatment with 0.1 µM BA-9G suppressed adventitious shoot formation (relative to the control). However, the number of adventitious shoots increased following the treatment with 1 µM BA-9G. Notably, 10 µM BA-9G induced adventitious shoot formation, which was in contrast to the fatal effect of BA at the same concentration, although the number of shoots was similar to that observed at 1 µM. These findings along with the observed effects on Pn cells suggest that BA-9G acts as a cytokinin with moderate biological activity and reduced cytotoxicity at high concentrations.

**Figure 3:**
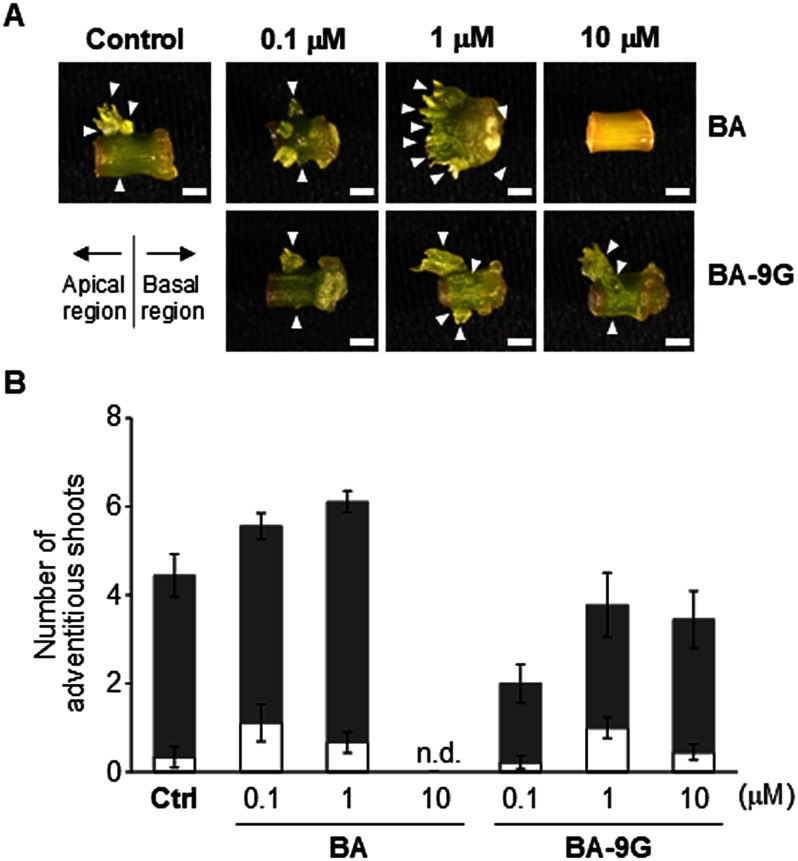
Figure 3. Effects of BA and BA-9G on adventitious shoot formation in ipecac internodes. (A) Representative images of internodal segments cultured for 5 weeks under each condition. Left and right halves of internodes indicate apical and basal regions, respectively. Arrowheads indicate adventitious shoots that formed. The control sample was treated with dimethyl sulfoxide, which was used as the solvent to dissolve BA and BA-9G. Scale bars are 2 mm. (B) Number of adventitious shoots formed in apical (filled bars) and basal (empty bars) regions. Data are presented as the mean±SE (*n*=9). Ctrl, control. n.d., not detected.

*N*-Glucosides of cytokinins have been identified in a wide variety of plant species (Hoyerová and Hošek 2020). Although the purine moiety can be glucosylated at the *N*^3^, *N*^7^, or *N*^9^ position, *N*^7^- and *N*^9^-glucosides are the predominant forms, whereas *N*^3^-glucosides are relatively rare (Chen et al. 2021; Hoyerová and Hošek 2020). The cytokinin activity of *N*^3^-glucosides markedly exceeds that of *N*^7^- and *N*^9^-glucosides, likely because *N*^3^-glucosides are more susceptible to the hydrolysis catalyzed by β-glucosidase to form the free base than *N*^7^- and *N*^9^-glucosides (Letham et al. 1975). Although *N*^7^- and *N*^9^-glucosides have historically been considered to be inactive or have low to negligible biological activity, a contrasting view has also been reported (Chen et al. 2021). It is still controversial whether *N*-glucosides are biologically active or if they need to be hydrolyzed to their free base (Chen et al. 2021; Hošek et al. 2020; Hoyerová and Hošek 2020). Despite this uncertainty, our results suggest that BA-9G may be useful as a plant growth regulator with moderate biological activity and reduced cytotoxicity (relative to free BA).

However, because of its limited availability, BA-9G is relatively expensive, which is not conducive to its widespread use. In this study, the maximum production titer of BA-9G per liter culture (set to 100% in Supplementary Figure S2D) was calculated to be approximately 50 mg; this was produced after only 4 days. BA-9G bioproduction using Pn cells may be better than chemical synthesis, which requires complex multistep reactions for regio-specific *N*-glucosylation (Cowley et al. 1978), in terms of simplicity and low environmental loads. This bioproduction system may increase the availability of BA-9G, a highly expensive and rare cytokinin derivative. Future studies should examine whether this system is also applicable to the bioproduction of *N*^9^-glucosides of natural cytokinins (e.g., *trans*/*cis*-zeatin, isopentenyl adenine, and dihydrozeatin) with isoprenoid-type *N*^6^-substituents.

According to earlier research, *N*^7^- and *N*^9^-glucosides of cytokinins are often found together (Chen et al. 2021; Hošek et al. 2020; Hoyerová and Hošek 2020). To the best of our knowledge, the accumulation of *N*^9^-glucoside unaccompanied by *N*^7^-glucoside, as observed in this study, is quite rare. Indeed, the regio-specific *N*-glucosyltransferase of cytokinins has not been reported (Hou et al. 2004). Considering that neither BA-7G nor BA-3G were found in the extract of BA-treated Pn cells, Pn cells likely contain an *N*^9^-specific glucosyltransferase. This enzyme is currently being analyzed in our laboratory.

## Abbreviations

BA: *N*^6^-benzyladenine
BA-3G: *N*^6^-benzyladenine *N*^3^-β-D-glucopyranoside
BA-7G: *N*^6^-benzyladenine *N*^7^-β-D-glucopyranoside
BA-9G: *N*^6^-benzyladenine *N*^9^-β-D-glucopyranoside
FW: fresh weight
HPLC: high-performance liquid chromatography
HR-ESI-TOF-MS: high-resolution electrospray ionization time-of-flight mass spectrometry
MS: Murashige and Skoog
Pn: *Phyllostachys nigra*
SCV: sedimented cell volume

## Description of supplementary files

Supplementary Methods. Isolation of compound 1 from Pn suspension cells.

Supplementary Figure S1. Effects of the initial BA concentration on cell proliferation and the production of compound 1 at an initial cell density of 5% SCV.

Supplementary Figure S2. Effects of the initial cell density on cell proliferation and the production of compound 1 at an initial BA concentration of 250 µM.

Supplementary Figure S3. HPLC chromatograms of extracts of Pn suspension cells before (day 0) and after (day 4) the culture with an initial cell density of 20% SCV in the presence of 250 µM BA.

Supplementary Figure S4. HR-ESI-TOF-MS spectrum of purified compound 1 (BA-9G).

Supplementary Figure S5. NMR spectra of purified compound 1 (BA-9G).

Supplementary Figure S6. HPLC chromatograms of purified compound 1 (BA-9G) and authentic standards of *N*-glucosides of BA (BA-3G, BA-7G, and BA-9G).

Supplementary Figure S7. Lignification profiles of Pn suspension cells cultured in the presence of 10 µM BA or BA-9G.
