# Supplementary material for: Bioproduction of N6-benzyladenine N9-β-D-glucopyranoside using suspension cells of bamboo (Phyllostachys nigra)
Source: Plant Biotechnol (Tokyo). 2025 Dec 25;42(4):485–9. doi: 10.5511/plantbiotechnology.25.0507a (PMC12781912; doi:10.5511/plantbiotechnology.25.0507a)
Supplement: Supplementary Data [file plantbiotechnology-42-4-25.0507a-s001.pdf]

Supplementary Information

for:

Bioproduction of *N*⁶-benzyladenine *N*⁹-β-D-glucopyranoside using suspension cells of bamboo (*Phyllostachys nigra*)

Taiji Nomura[1,*], Karin Okazaki[2,a], Mikihisa Umehara[2], Yasuo Kato[1]

[1]*Biotechnology Research Center and Department of Biotechnology, Toyama Prefectural University, 5180 Kurokawa, Imizu, Toyama 939-0398, Japan*

[2]*Graduate School of Life Sciences, Toyo University, 48-1 Oka, Asaka, Saitama 351-8510, Japan*

[a]*Present address: Center for Sustainable Resource Science, RIKEN, 1-7-22 Suehiro-cho, Tsurumi-ku, Yokohama, Kanagawa 230-0045, Japan*

[*]Corresponding author e-mail: tnomura@pu-toyama.ac.jp

Supplementary Methods

Isolation of compound 1 from Pn suspension cells

Pn suspension cells were cultured for 4 days in 1/2 MS medium supplemented with 250 µM BA, with an initial cell density of 20% SCV. Cells from a 2.3-l culture (100 ml × 23) were collected on filter paper via vacuum filtration. The collected cells (187 g FW) were mixed with 1.5 l MeOH containing 2% (v/v) AcOH for a 1-h ultrasonication at room temperature. The resulting extract was passed through filter paper and then the filtrate was concentrated and defatted by washing four times with *n*-hexane. The aqueous layer was concentrated to a small volume, which was subsequently applied to an octadecylsilyl (ODS) column (Cosmosil 75C18-OPN; Nacalai Tesque, Kyoto, Japan; 4 × 32 cm; 400 ml column volume) equilibrated with water. The following solvents (2 l each) were used for the sequential elution from the column: 0%, 20%, 40%, 60%, 80%, and 100% (v/v) MeOH. The eluate derived from the elution using 40% MeOH was concentrated to a small volume, passed through a membrane filter (Millex-HV, 0.45 µm; Merck, Darmstadt, Germany), and subjected to two rounds of reversed-phase preparative HPLC (column, TSKgel ODS-80Ts; 5 µm, 20 × 250 mm; Tosoh, Tokyo, Japan; solvent, 40% (v/v) MeOH containing 0.1% (v/v) trifluoroacetic acid; flow rate, 5 ml min^{-1}; detection wavelength, 300 nm). The collected fraction was concentrated and lyophilized to obtain compound 1 (122 mg white powder as trifluoroacetic acid salt). A portion of the purified trifluoroacetic acid salt was converted to the free amine form for a comparison of the NMR spectral data with those reported in the literature. More specifically, 20 mg purified compound was dissolved in 10 ml water, neutralized with 0.1 M NaOH, and applied to a Sep-Pak Plus C18 Cartridge (1 ml; Waters, Milford, MA, USA). The cartridge was washed twice with 10 ml water prior to the elution using 10 ml MeOH. The eluate was concentrated and lyophilized to obtain compound 1 (12.4 mg white powder as free amine form).

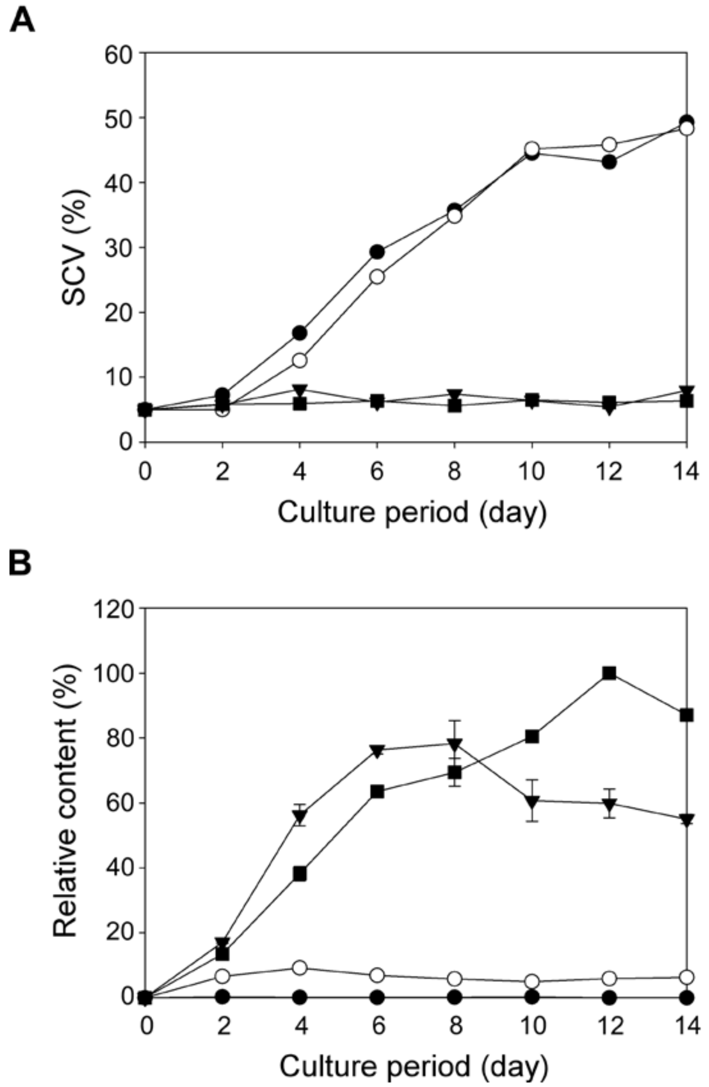

Supplementary Figure S1. Effects of the initial BA concentration on cell proliferation and the production of compound 1 at an initial cell density of 5% SCV. (A) Changes in SCVs (n = 1). (B) Changes in the compound 1 content (mean ± SD, n = 3). The content calculated as the HPLC peak area per mg FW was compared by setting the maximum value to 100%. 1 μM (filled circles), 10 μM (empty circles), 100 μM (filled triangles), and 250 μM (filled squares).

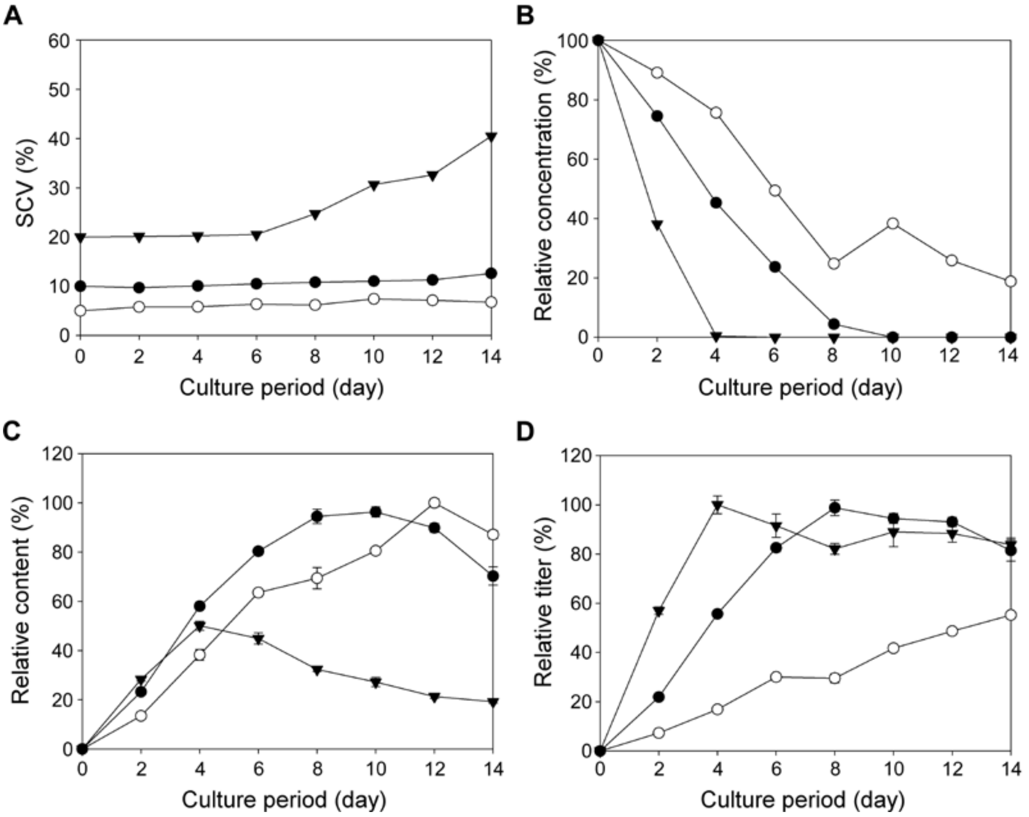

Supplementary Figure S2. Effects of the initial cell density on cell proliferation and the production of compound 1 at an initial BA concentration of 250 µM. (A) Changes in SCVs (*n* = 1). (B) Changes in the BA concentration in the culture medium (*n* = 1). The initial concentration (250 µM) was set to 100%. (C) Changes in the compound 1 content (mean ± SD, *n* = 3). The content calculated as the HPLC peak area per mg FW was compared by setting the maximum value to 100%. (D) Changes in the production titer per liter culture (mean ± SD, *n* = 3). The content calculated as the HPLC peak area per liter culture was compared by setting the maximum value to 100%. 5% (empty circles), 10% (filled circles), and 20% (filled triangles) SCVs.

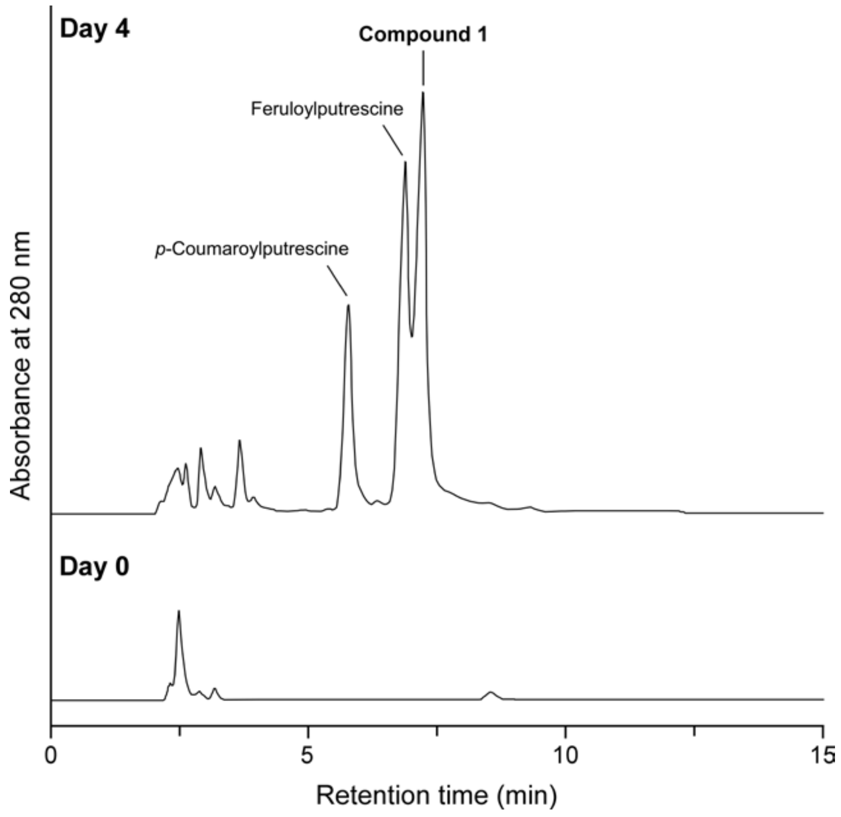

Supplementary Figure S3. HPLC chromatograms of extracts of Pn suspension cells before (day 0) and after (day 4) the culture with an initial cell density of 20% SCV in the presence of 250 μM BA. In addition to compound 1, hydroxycinnamoylputrescines (i.e., *p*-coumaroylputrescine and feruloylputrescine) were observed in the cells cultured in the presence of BA as we previously described (Nomura et al. 2013).

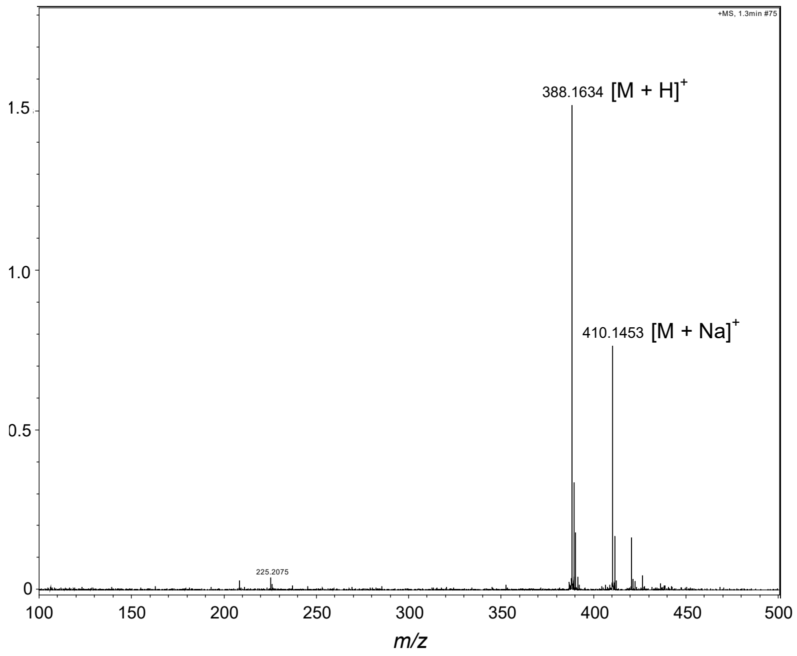

Supplementary Figure S4. HR-ESI-TOF-MS spectrum of purified compound 1 (BA-9G).

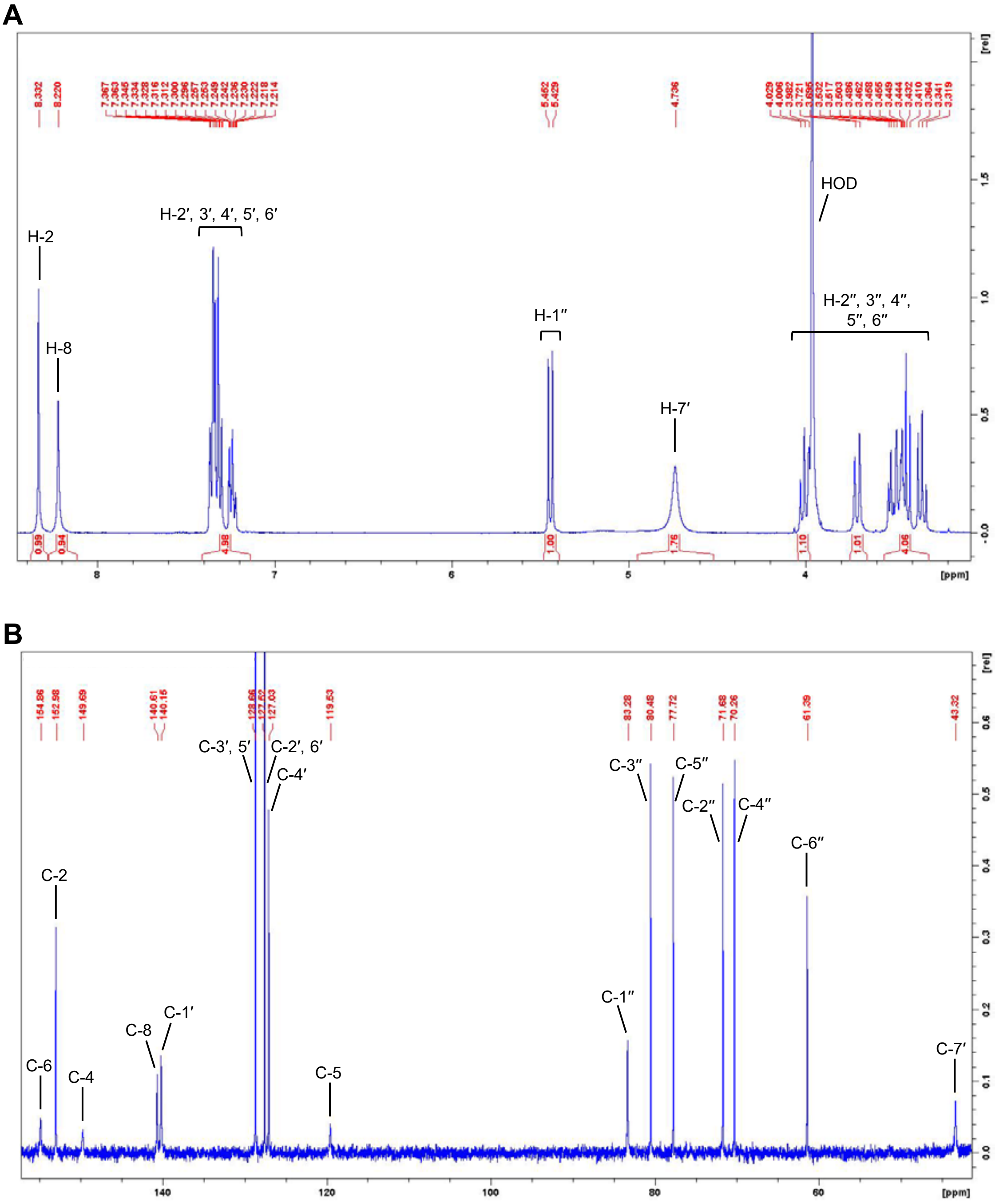

Supplementary Figure S5. NMR spectra of purified compound 1 (BA-9G). (A) ^{1}H-NMR spectrum (400 MHz, $(CD_3)_2SO/D_2O$, 5/1). (B) ^{13}C-NMR spectrum (100 MHz, $(CD_3)_2SO$).

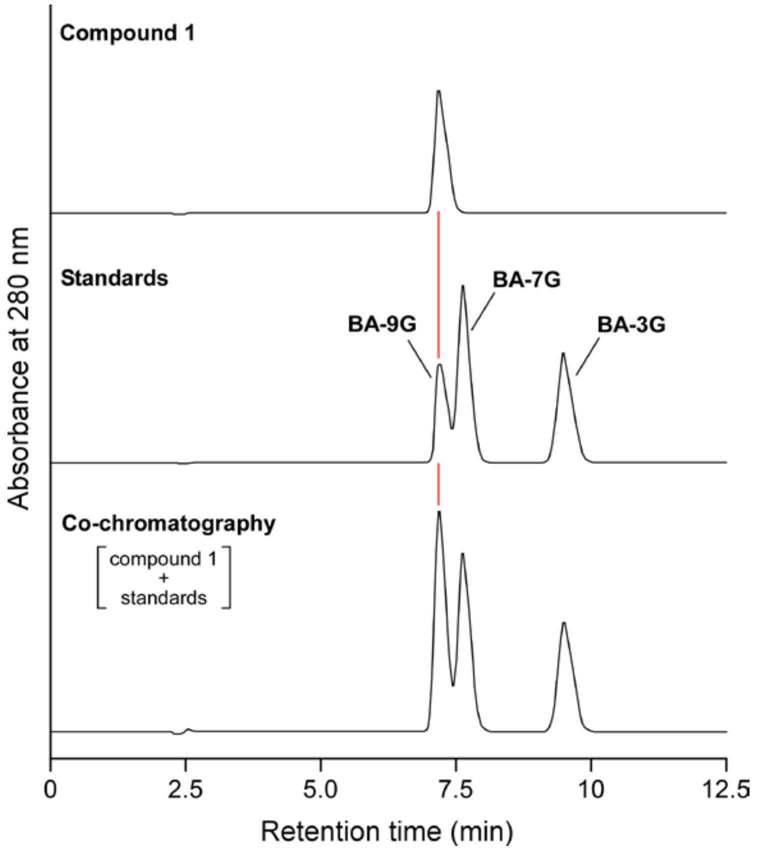

Supplementary Figure S6. HPLC chromatograms of purified compound 1 (BA-9G) and authentic standards of *N*-glucosides of BA (BA-3G, BA-7G, and BA-9G).

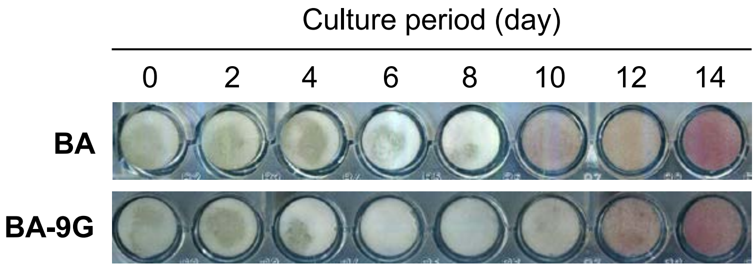

Supplementary Figure S7. Lignification profiles of Pn suspension cells cultured in the presence of 10 μM BA or BA-9G. Some of the cells collected at each time point were fixed and stained with a phloroglucinol–HCl (Wiesner) reagent in a 96-well microplate as previously described (Ogita et al. 2012).

References for Supplementary Information

Nomura T, Shiozawa M, Ogita S, Kato Y (2013) Occurrence of hydroxycinnamoylputrescines in xylogenic bamboo suspension cells. *Plant Biotechnol (Tokyo)* 42: 65–72

Ogita S, Ohki S, Nomura T, Kato Y (2012) A β-glucosidase activity potentially involved in cell division and wall development of *Phyllostachys* bamboo suspension cells. *Am J Plant Sci* 3: 1066–1072
